# Supplementary material for: An Unconventional Case Study of Neoadjuvant Oncolytic Virotherapy for Recurrent Breast Cancer
Source: Vaccines (Basel). 2024 Aug 23;12(9):958. doi: 10.3390/vaccines12090958 (PMC11435696; doi:10.3390/vaccines12090958)
Supplement: Supplementary file 1 [file vaccines-12-00958-s001.zip › vaccines-3147363-Supplementary Material.pdf]

# Detailed description of case history

### First diagnosis

The tumour was first diagnosed in 2016 after sudden (overnight) swelling of the whole breast, which was treated surgically (mastectomy) followed by adjuvant chemotherapy with doxorubicin and cyclophosphamide (4 cycles with 60 and 600 $mg/m^2$, respectively). Histopathological analysis of excised breast revealed several foci of invasive ductal cancer (largest 0.7 cm) negative for oestrogen receptors, progesterone receptors and HER2 (ER 0, PR 0, HER2 0), while the 80% of the tumour mass contained *in situ* cancer of the same triple negative phenotype. The tumour was of histological grade III with lymphovascular (LVI) and perineural (PNI) invasion, with proliferation index Ki67 55%, and was negative for androgen receptor, CK 5/6, and CK 14.

### First recurrence

In 2018 a small local recurrence at the site of the previous surgery (below the suture) was detected and surgically removed (TNBC, grade III, 0.4 cm, ER 0, PR 0, HER2 0, LVI and PNI negative, Ki67 23%). No adjuvant therapy was administered per the wish of the patient. However, a small seroma (<1 cm) remained at the site of excision and this was periodically monitored.

### Second recurrence

Phase contrast magnetic resonance imaging (MRI) in August 2020 (as part of a regular monitoring protocol) showed that the structure so far described as "seroma" had progressed to become a 2 cm diameter solid tumour (in January 2020 the size of "seroma" was estimated to 1.2 cm by ultrasound and mammography). The patient, who is also a virologist (1-7) and anticipating that the recurrent tumour would be of TNBC phenotype for which no specific and effective therapy exists, informed her oncologists that she was going to try to treat this tumour by the *i.t.* administration of viruses similar to the oncolytic viruses (OVs) that are in clinical development for breast cancer, before trying any other treatment. Her oncologists accepted to monitor the progress of the treatment, mainly with the aim to stop the injections and intervene with conventional therapy if there were untoward effects or if the tumour progressed. The oncolytic virotherapy (OVT) started immediately after all necessary baseline diagnostic tests had been run, including FDG PET-CT scan and core needle biopsy sampling of the tumour. However, at the time of OVT initiation the tumour tissue had not yet been analysed. Surprisingly, histopathological analysis of core biopsy material subsequently indicated that the tumour had evolved from TNBC to HER2 3+ (grade III, ER 0, PR 0, HER2 3+, Ki-67 45%). The oncologists therefore suggested to the patient a neoadjuvant therapy combining chemotherapy with trastuzumab and pertuzumab prior surgical removal of the tumour and continuation with anti-HER2 monoclonal antibodies up to one year, a therapy known to have significant efficacy in treating HER2 3+ tumours. However, by this time the patient was already experiencing some clinical benefit from the OV therapy and decided to continue with her unconventional OVT protocol until the disease progression (which has not yet occurred).

At baseline the recurrent tumour was described as a hard, palpable nodule on the chest wall, at the site of previous mastectomy, bellow the surgical suture from the previous operations. The tumour was further described as being bright red, inflamed, and with a thin skin above it. Different imaging techniques described it as a circled plate at the base (chest wall side), with a bulging hill at the skin side. MRI, PET-CT scan, and two independent ultrasound estimations all gave matching tumour volume estimations of 2.47 ± 0.06 $cm^3$. MRI showed that the tumour had invaded into the pectoral muscle and this was supported by the PET-CT. Skin infiltration was identified on all three of the diagnostic imaging methods. Under ultrasound, the tumour appeared as a hypoechoic and spiculated mass. PET-CT scan showed no evidence for metastatic disease or local spread to regional lymph nodes.

**Detailed follow-up on OVT**

The therapy was started with *i.t.* MeV application. MeV was applied 7 times at three- to four-day intervals over a period of three weeks, in the cumulative quantity of $10^{7.9}$ $CCID_{50}$ (Figure 1B). There were no systemic side effects. Due to extreme hardness of the tumour mass, the needle insertion and virus suspension injection in the first three application was difficult. Minute amounts of suspension were, therefore, applied at multiple sites and the administration was moderately painful. It was accompanied with prolonged bleeding from the injection site (dark, capillary venous blood). One week after the first MeV administration (before the third dose) the tumour mass had enlarged and was tense, with extremely thin skin covering the mass and looking like it might break. Under ultrasound the mass appeared more hypoechoic and more spiculated compared to baseline, and was slightly larger. The first sign of improvement was observed on the day of 11 ($4^{th}$ MeV). The clinical picture was improved with less redness and less tension. Under ultrasound the mass appeared less spiculated and better circumscribed. The majority of the mass was soft, and needle insertion and virus application were easier, no longer painful and not accompanied by bleeding from the injection site. It was the first time when 2 mL of suspension could be easily administered into the tumour. From that time until the last ($7^{th}$) MeV administration on day 21, the mass was constantly shrinking by palpation and was less tense, with less overlying redness (it looked more like a healing bruise). Also, the mass was getting better circumscribed, less spiculated and less hypoechoic on ultrasound (Figure 2A) and *i.t.* virus administration was becoming steadily easier.

Three days after the $7^{th}$ MeV application, VSV therapy was initiated. The tumour was not dense any more, the needle was easily inserted, and a higher volume of virus suspension was easily administered, in comparison to the first MeV inoculation. Two mL of VSV suspension were distributed at multiple intratumoural foci. A few hours later, the tumour was obviously swollen and the skin was very red. Twelve hours after the administration, the patient developed rigors that lasted for 2 hours and became febrile to 40 °C. The high fever persisted for approximately 24 hours despite ibuprofen therapy and resolved slowly during the second day post virus administration. This was the moment when the tumour was of the highest softness (like a sponge) during the whole OVT period. Because of the strong side reaction to the first dose of VSV, additional dosing was temporarily suspended and a phase-contrast MRI was performed two weeks later. While the repeated MRI did not at this time point show substantial reduction in the size of the mass, there was no longer any visible infiltration of the underlying pectoral muscle. An increased number of enlarged lymph nodes was observed in both axillae compared to the baseline MRI. The patient was then referred to a surgeon who noted that the tumour mass was now freely mobile over the underlying muscle and could be easily removed by surgery. Prior to the planned date of surgery, the VSV administration was continued in one-week intervals. In the second administration, only 1 mL was applied as a precautionary measure to reduce potential systemic side effects, but this time, there was no febrile reaction, nor was there any adverse reaction after the last administration of 1.1 mL of VSV given a week later. At that time, the tumour had a more cystic appearance by ultrasound scan. In total, three doses of VSV were administered, separated by two and one weeks, respectively, within the total period of 24 days. The cumulative dose of administered VSV was $10^{9.1}$ $CCID_{50}$, and volumes and infective virus quantities in separated doses are listed in Figure 1B.

Ten days after the last (the third) VSV application, the tumour was excised. Two months after the tumour excision, MeV was applied subcutaneously once around the surgical suture, as a preventive adjuvant treatment.

**Detailed materials and methods**

*Oncolytic viruses*

Edmonston Zagreb measles vaccine strain (Institute of Immunology Inc., Zagreb, Croatia) (8-9) and vesicular stomatitis virus Indiana strain (ATCC) were used for laboratory-grade production of viruses for OVT. The genome sequence of Edmonston Zagreb MeV has been determined and deposited in GenBank under accession numbers AY486084. Working banks of Vero and MRC-5 cells, that originate from Vero (WHO) (ECACC 88020401) and MRC-5 pd19 (ECACC 05072101), respectively, were provided by the Institute of Immunology Inc. The registered production process of pediatric measles vaccine (Institute of Immunology Inc.) was scaled down for laboratory-grade production of both viruses for this study.

MeV was propagated in MRC-5 or Vero cell culture (as indicated in Figure 1B). Infections were performed by Edmonston Zagreb vaccine strain working seed (EZ D242/99, Institute of Immunology Inc.) at a cell density of 75,000 cells/$cm^2$ when MRC-5 or 100,000 cells/$cm^2$ when Vero cells were used with a MOI of 0.01 in MEM + 10% FBS. After 24 hours of cultivation at 36 °C, the medium was replaced with MEM without FBS, and cultivated at 32 °C, 5% $CO_2$ until the appearance of cytopathic changes. Supernatant of such cell culture was collected, clarified by centrifugation (10 min, 1400 *g*) and sterilized by 0.45 μm filtration (Millipore).

VSV was propagated in Vero cell culture. Infections were performed by Laboratory VSV working seed at a cell density of 100,000 cells/$cm^2$, with a MOI of 0.5 in MEM +10% FBS. After 4 hours of cultivation at 37 °C, the medium was replaced with MEM without FBS and cultivated for 24 hours and 37 °C, 5% $CO_2$, until the appearance of cytopathic changes. Virus suspensions were collected from the cell culture supernatant after centrifugation (10 min, 1400 *g*) and were sterilized by 0.45 μm filtration (Millipore).

Freshly prepared virus suspension was used for each OVT application and time between the virus harvest and administration was never longer than one hour. Each time an aliquot of suspension was analysed by $CCID_{50}$ assay for precise determination of the exact infective virus concentration in each applied dose.

*50% cell culture infective dose ($CCID_{50}$) assay*

MeV and VSV titres were determined using the standard $CCID_{50}$ assay in 96-well format (2). Octaplicates of three-fold serial dilutions of virus suspension (100 μL) were mixed with Vero cell suspension (100 μL) ($2 \times 10^5$/mL or $1.2 \times 10^5$/mL in MeV assay). Each sample was investigated in a suitable range of serial dilutions resulting from 100 to 0% CPE readings. After four (VSV assay) or six (MeV assay) days of incubation at 37 °C and 5% $CO_2$, wells with CPE were counted and $CCID_{50}$/mL calculated using Spearman-Kärber method. Virus titers were expressed as a logarithm of a reciprocal value of a dilution causing 50% of CPE and calculated using the Spearman-Kärber formula: $\log_{10}CCID_{50} = L - d/(S - 0.5)$ where $L = \log_{10}$ starting sample dilution, $d = \log_{10}$ dilution step, S = sum of the proportion of positive replicate. In each run the assay was in parallel performed with in-house standard sample of respective virus with established nominal value. Any deviation of the standard sample result from its nominal value was used for correction of results of experimental samples in the respective assay run.

*Assessment of response to oncolytic virotherapy*

The changes in visual clinical presentation were described and photographed. Before the OVT, the size of the tumour was estimated by PET-CT scan, phase contrast magnetic resonance imaging (MRI) and two ultrasound scans performed using two different systems operated by two independent radiologists. During the therapy, changes in tumour size and appearance were regularly and very often monitored using ultrasound imaging. Phase contrast MRI was performed once during the course of therapy, and the size of the tumour mass was determined at the end of therapy by histopathologist examining the excised tumour. The tumour size was estimated as the product of the length, height and width, as if it was a regular hexahedron and expressed in $cm^3$.

*Antivirus antibody titres*

Anti-MeV and anti-VSV neutralizing antibody titres (NT) were determined using a standard effective dose 50 ($ED_{50}$) assay in a 96-well format (1,2,7). Briefly, two-fold serial dilutions of patient's serum samples taken at different time points during the therapy, each in octaplicate (50 µL/well) were preincubated with approximately 20 $CCID_{50}$ (50 µL/well) of the respective virus at 37 ºC and 5% $CO_2$ for 90 minutes. After the addition of Vero cells ($1.2 \times 10^5$/mL for anti-MeV and $2 \times 10^5$/mL for anti-VSV assay; 100 µL/well), the plates were incubated at 37 °C and 5% $CO_2$. The wells with pure cell suspension served as cell growth control. After incubation period (6 days-long for anti-MeV and 4 days-long for anti-VSV assay), the wells with cytopathic changes visible under inverted optical microscope were counted, and the $ED_{50}$ (the amount of undiluted serum that inhibits the cytopathic effect in 50% of infected wells) calculated according to the Spearman-Kärber method. NT was expressed as the number of $ED_{50}$s in 1 mL of serum.

The IVIg preparation (Institute of Immunology Inc., Zagreb, Croatia) was calibrated against 3rd WHO International Standard for Anti-Measles (97/648), and used as in-house reference material for determination of anti-MeV NT.

*Pathohistological and immunohistochemistry characterization of excised tumour*

Tumour tissue samples were fixed in 10% buffered formalin for 48 hours, after which the tissue was embedded in paraffin (routine FFPE procedure). Before the immunohistochemical staining (IHC) procedure, the pathologist re-examined the histological slides and determined a representative slide for further analysis. Several 3-5 µm thick sections were cut from the selected paraffin block, and placed on positively charged glass slides for IHC. Two staining protocols were used for IHC staining, which have the same HRP/DAB staining principle, and the details depend on the visualization kit and the device in which the staining was performed.

The first IHC protocol was performed in an automated Dako Autostainer Link48 device using the EnVisionTM FLEX/HRP high pH detection kit containing all reagents required for staining (cat. no. K8000; Dako, Denmark). Antigen retrieval on tissue sections was performed by heat treatment in the PTLink module using Dako 3 in 1 Antigen retrieval buffer pH 9.0. To visualize specific immune cell markers, the following primary antibodies were applied to tumor tissue for 30 minutes at room temperature: CD3 (clone F2.2.38, Dako; dilution 1:100); CD4 (clone 4B12, Dako; dilution 1:100); CD8 (clone C8/144B, Dako; dilution 1:100); CD20cy (clone L26, Dako; dilution 1:100); CD56 (clone 123C3, Dako; dilution 1:100), and CD68 (clone KP1, Dako; dilution 1:100). After washing with wash buffer, the secondary antibody was applied for 30 minutes at room temperature. After repeating the washing step again, DAB chromogen was applied for 10 minutes and then contrast staining with haematoxylin. The determination of the percentage of IHC-positive immune cells was performed on the entire tissue section.

The second IHC staining protocol for PD-L1 was performed in a fully automated Ventana BenchMark GX® device using the primary anti-PD-L1 antibody clone SP142 (ref. no. 741-4860, Ventana) and the secondary visualization antibody contained in the OptiView DAB IHC Detection kit (ref. no. 760-700, Ventana) according to a protocol routinely used in triple-negative breast cancer. The specific scoring of the PD-L1 reaction was done according to the criteria for triple-negative breast cancer, where only intratumoural or peritumoural areas are considered, while distant immune cells are not counted.

Representative photomicrographs of immunohistochemistry slides were captured using Zeiss ZEN 2.3 Lite light microscope imaging software.

## References


1. Halassy, B.; Kurtović, T.; Brgles, M.; Lang Balija, M.; Forčić, D. Factors influencing preclinical in vivo evaluation of mumps vaccine strain immunogenicity. *Hum. Vaccines Immunother.* **2015**, *11:* 2446–2454. https://doi.org/10.1080/21645515.2015.1062191.
2. Brgles, M.; Kurtović, T.; Lang Balija, M.; Hećimović, A.; Mušlin, T.; Beata H. Impact of complement and difference of cell-based assay and ELISA in determination of neutralization capacity against mumps and measles virus. *J. Immunol. Methods.* ***2021***, 490: 112957. https://doi.org/10.1016/j.jim.2021.112957.

3. Kutle, L.; Pavlović, N.; Dorotić, M.; Zadro, I.; Kapustić, M.; Halassy, B. Robustness testing of live attenuated rubella vaccine potency assay using fractional factorial design of experiments. *Vaccine.* ***2010***, 28: 5497–5502. https://doi.org/ 10.1016/j.vaccine.2010.04.111.
4. Brgles, M.; Halassy, B.; Forčić, D. An elution mobile phase and process for immunoaffinity chromatography of viruses. Patent EP003408383B1. Published 18 December 2019. p. 25 pp. https://worldwide.espacenet.com/patent/search/family/058009779/publica-tion/EP3408383B1?q=pn%3DUS10597640B2
5. Brgles, M.; Halassy, B.; Forčić, D. Elution mobile phase and process for immunoaffinity chromatography of viruses. Patent US10597640B2. Published 24 March 2020. 15 pp. https://worldwide.espacenet.com/patent/search/family/058009779/publication/US10597640B2?q=Halassy
6. Brgles, M.; Sviben, D.; Forčić, D.; Halassy, B. Nonspecific native elution of proteins and mumps virus in immunoaffinity chromatography. *J. Chromatogr. A*. ***2016***, 1447: 107–114. https://doi.org/10.1016/j.chroma.2016.04.022.
7. Ravlić, S.; Hećimović, A.; Kurtović, T.; Ivančić Jelečki, J.; Forčić, D.; Slović, A.; Kurolt, I.C.; Šafranko, Ž.M.; Mušlin, T.; Rnjak, D.; et al. Is Better Standardization of Therapeutic Antibody Quality in Emerging Diseases Epidemics Possible? *Front. Immunol*. ***2022***, 13: 816159. https://doi.org/10.3389/fimmu.2022.816159.
8. Ikić, D.; Juzbasić, M.; Beck, M.; Hrabar, A.; Cimbur-Schreiber, T. Attenuation and characterization of Edmonston-Zagreb measles virus. *Ann. Immunol. Hung.* ***1972***, 16: 175-81.
9. Baričević, M.; Forčić, D.; Košutić Gulija, T.; Jug, R.; Mažuran, R. Determination of the coding and non-coding nucleotide sequences of genuine Edmonston–Zagreb master seed and current working seed lot. *Vaccine.* ***2004***, 23: 1072–1078. https://doi.org/10.1016/j.vaccine.2004.08.021.
